# Material Optimization Engineering toward xLiFePO_4_·yLi_3_V_2_(PO_4_)_3_ Composites in Application-Oriented Li-Ion Batteries

**DOI:** 10.3390/ma15103668

**Published:** 2022-05-20

**Authors:** Yuqiang Pi, Gangwei Luo, Peiyao Wang, Wangwang Xu, Jiage Yu, Xian Zhang, Zhengbing Fu, Xiong Yang, Li Wang, Yu Ding, Feng Wang

**Affiliations:** 1 School of Chemistry and Materials Science, Hubei Engineering University, Xiaogan 432000, China; xq0502@163.com (Y.P.); 15885400892@163.com (G.L.); yujiage5@163.com (J.Y.); zxian821@163.com (X.Z.); ceramic425@163.com (Z.F.); yangx@hbeu.edu.cn (X.Y.); wangli19810609@126.com (L.W.); 2 Department of Mechanical Engineering, The University of Melbourne, Parkville, VIC 3010, Australia; peiyao.wang@student.unimelb.edu.au; 3 College of Materials and Chemical Engineering, China Three Gorges University, Yichang 443002, China; wxu26@lsu.edu; 4 Qinghai Electronic Material Industry Development Co., Ltd., Xining 810006, China

**Keywords:** xLiFePO_4_·yLi_3_V_2_(PO_4_)_3_, spray-drying, power capacity, compacted density, Li-ion batteries

## Abstract

The development of LiFePO_4_ (LFP) in high-power energy storage devices is hampered by its slow Li-ion diffusion kinetics. Constructing the composite electrode materials with vanadium substitution is a scientific endeavor to boost LFP’s power capacity. Herein, a series of xLiFePO_4_·yLi_3_V_2_(PO_4_)_3_ (xLFP·yLVP) composites were fabricated using a simple spray-drying approach. We propose that 5LFP·LVP is the optimal choice for Li-ion battery promotion, owning to its excellent Li-ion storage capacity (material energy density of 413.6 W·h·kg^−1^), strong machining capability (compacted density of 1.82 g·cm^−3^) and lower raw material cost consumption. Furthermore, the 5LFP·LVP||LTO Li-ion pouch cell also presents prominent energy storage capability. After 300 cycles of a constant current test at 400 mA, 75% of the initial capacity (379.1 mA·h) is achieved, with around 100% of Coulombic efficiency. A capacity retention of 60.3% is displayed for the 300th cycle when discharging at 1200 mA, with the capacity fading by 0.15% per cycle. This prototype provides a valid and scientific attempt to accelerate the development of xLFP·yLVP composites in application-oriented Li-ion batteries.

## 1. Introduction

The commitment to carbon neutrality and the implementation of a low-carbon economy in the world have become the key to the sustainable development of human society, given the massive population explosion and incremental demand for new or renovated infrastructure [1,2]. The most reliable technique for reducing carbon emissions is to increase the utilization of available renewable energy sources [3,4,5]. The energy storage technologies (ESTs) are critical to attaining renewable energy sources with intermittent and unstable characteristics. Among the variety of ESTs, lithium-ion batteries (LIBs) have been widely recognized in the daily life of human beings, due to their high-energy density and ultralong lifespan [6,7,8].

Cathode material as a key component determines many aspects of LIBs, including the Li-ion storage capability, lifespan, cost, and safety [9,10]. In particular, olivine LiFePO_4_ (LFP) has received substantial attention from academia and industry, because of its relatively high theoretical energy density (580 W·h·kg^−1^), structural integrity during the Li-ion extraction/insertion process, and low raw cost consumption [11,12]. At present, LFP research is primarily focused on three aspects: (i) increasing discharge specific capacity, (ii) enhancing the power capability, and (iii) improving the low-temperature performance. In particular, the sluggish Li-ion diffusion kinetics of LFP impedes its development in high-power energy storage devices [13,14]. Many attempts have been made to favor Li^+^ fast migration in order to address this issue. One of them is to modify LFP by combining it with Li_3_V_2_(PO_4_)_3_ (LVP), which has three-dimensional pathways for Li^+^ mobility [15,16]. However, such a strategy proposed by many researchers mainly aims at improving electrochemical performance, leaving out application-oriented research on the compacted density and Li-ion pouch cells [17,18,19,20].

In addition to selecting a cathode material with a high Li-ion diffusion coefficient, pursuing high-power LIBs is still dependent on several factors. Firstly, the anode material should be carefully considered. Li_4_Ti_5_O_12_ (LTO) is an appealing anode candidate for high-power LIBs, because of its fast Li^+^ transport capability, zero volume change during charging cycles, and alleviated lithium deposition [21,22]. Secondly, the component ratio of electrode pieces can be fine-tuned to adapt to rapid Li-ion intercalation and deintercalation behavior. Moreover, the use of high-conductivity additive, such as carbon nanotubes, can significantly improve the electron transfer capability of electrode pieces. Thirdly, another strategy is to replace traditional foils with new high-performance foils. For example, carbon-coated Al foils enable lower internal resistance, decreased polarization, and improved adhesion between active materials and the current collector. They provide an opportunity for LIBs to achieve a higher power density with a considerable energy density. In practice, the compacted density is a critical parameter of battery design, and the volumetric energy density of the battery can be indicated directly by the compacted density of electrode pieces, which is determined by the size distribution of active material [23,24], the components of electrode pieces, and the imposed pressure from roller machine [25].

To develop an application-oriented energy storage device with a high power density, material engineering optimization was conducted to manufacture a series of xLFP·yLVP composites including LFP·LVP, 3LFP·LVP, 5LFP·LVP, and 8LFP·LVP utilizing a simple spray-drying approach. 5LFP·LVP (5LFVP) stands out as a strong candidate cathode material for the high-power LIBs, due to its comprehensive superiorities of Li-ion storage capability, machining capability, and raw material cost consumption. Subsequently, a 5LFVP||LTO Li-ion pouch cell was exploited to demonstrate its significant energy storage capability. According to the characterization of Li-ion extraction/insertion capability, this prototype Li-ion full cell presents a reasonable and scientific attempt to promote the development of xLFP·yLVP composites in application-oriented Li-ion batteries.

## 2. Materials and Methods

The information on the crystallographic structure of synthetic products was obtained using a D8 Advance X-ray diffractometer (XRD, Bruker AXS, Karlsruhe, Germany) with a non-monochromatic Cu K α X-ray source. The features of amorphous carbon in xLFP·yLVP composites were determined using the Renishaw INVIA micro-Raman spectroscopy system (Gloucestershire, UK). The Vario EL cube CHNSO elemental analyzer (Hanau, Germany) was employed to examine the carbon content. The information of the valence state was evaluated by XPS measurement via an Ultra DLD with a monochromic Al X-ray source. The Microtrac S3500 laser (Microtrac Inc., Largo, FL, USA) particle size analyzer was used for the particle distribution. To determine the morphology of xLFP·yLVP, the JEOL-7100F (JEOL Ltd., Akishima, Tokyo, Japan) with energy-dispersive spectroscopy (Oxford EDS IE25, Oxford, UK) was employed. The detailed structural information was obtained using a JEM-2100F microscope (JEOL Ltd., Akishima, Tokyo, Japan).

## 3. Electrochemical Characterization

Li-ion half cells were assembled for estimating the difference in Li-ion storage capabilities between various xLFP·yLVP composites. In this study, the Li-ion half cells were divided into five components: Li foil, power-type electrolyte, separator films from Celgard company, 2025 coin-type battery shell, and electrode piece with 14 mm diameter. Each electrode piece had a weight ratio of 80:10:10, corresponding to xLFP·yLVP active materials, conductive carbon nanotube paste (5 wt.%, Shandong Chenghe New Material Co., Ltd., Zibo, China) and PVDF binder, respectively. The current collector was made of carbon-coated aluminum foil. The mass loading of all xLFP·yLVP electrodes was controlled between 2.0 and 3.0 mg·cm^−2^. The LAND CT2001A battery test systems were used to characterize the electrochemical performance. To evaluate the electrochemical behavior, cyclic voltammetry (CV) curves were collected using a CHI 760e electrochemical workstation. To achieve a reasonable pouch-cell capacity design, the half cells of LTO||Li were also assembled using the same method described above to assess the specific capacity and cyclability of anode material.

The pouch-cell fabrication allows a rational way to demonstrate the utilization of xLFP·yLVP composites in application-oriented Li-ion batteries. To meet the requirements of practical application, the ratio of electrode materials, for all types of xLFP·yLVP cathodes and LTO anode, was set to 90.5 wt.%. The contents of carbon nanotube conductive paste, super P, and PVDF binder in the pouch cells were adjusted subsequently to 1.5 wt.%, 2 wt.%, and 6 wt.%, respectively. Rolls of separator films were used to prevent direct contact between the cathode and anode pieces. Here, we proceeded with the control on a capacity ratio of the anode to cathode, which could fluctuate within the range 1–1.05. Moreover, the roller machine with 15 tons of pressure was performed to achieve the appropriate compacted densities of all types of xLFP·yLVP cathodes and LTO anode pieces. A battery test system of TITANS THCX-IO/5-96-M-A was used to estimate the electrochemical property of pouch cells.

## 4. Synthetic Procedure

**Scheme 1:**
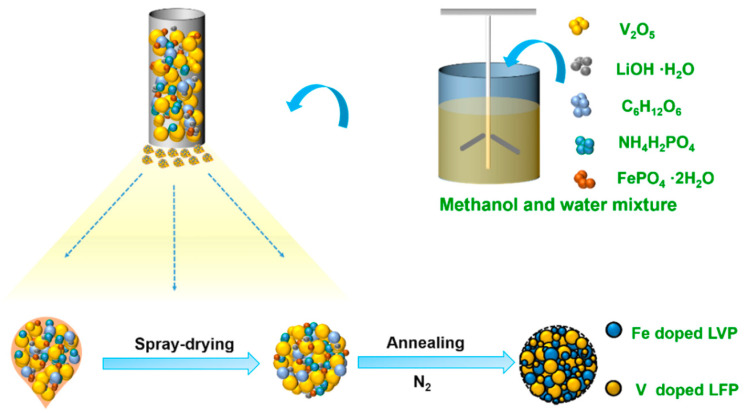
Schematic representation for manufacturing xLFP·yLVP composites.

In this work, material engineering optimization was conducted to manufacture the xLFP·yLVP composites using a simple spray-drying approach. Here, the molar ratio of x/y was varied from 1:1, 3:1, 5:1, to 8:1 with corresponding products denoted as LFVP, 3LFVP, 5LFVP, and 8LFVP, respectively. Scheme 1 depicts the detailed synthesis procedure of 3LFVP, which was chosen as a representative.

A sand-milling machine was operated to make all solid raw materials, i.e., 0.328 kg of V_2_O_5_, 1.009 kg of FePO_4_·2H_2_O, 0.465 kg of LiOH·H_2_O, 0.621 kg of NH_4_H_2_PO_4_, 0.5 kg of C_6_H_12_O_6_, and the milling solvent (10 L of methanol and 5 L of water) was mixed to form a uniform precursor liquid. The milling time was set at 2 h. The resulting slurry was then transformed into the precursor powder via the continuous heating exchange from the spray-drying equipment. Finally, the abovementioned precursors were sintered in N_2_ atmosphere at 700 °C for 12 h, allowing consistent xLFP·yLVP composites. Other xLFP·yLVP composites could be obtained by adjusting the ratio of V_2_O_5_ to FePO_4_·2H_2_O in the same synthesized procedure. Moreover, according to the theoretical content calculation of LFP and LVP in xLFP·yLVP composites (Table S1), the components could be recognized. 

## 5. Results and Discussion

**Figure 1:**
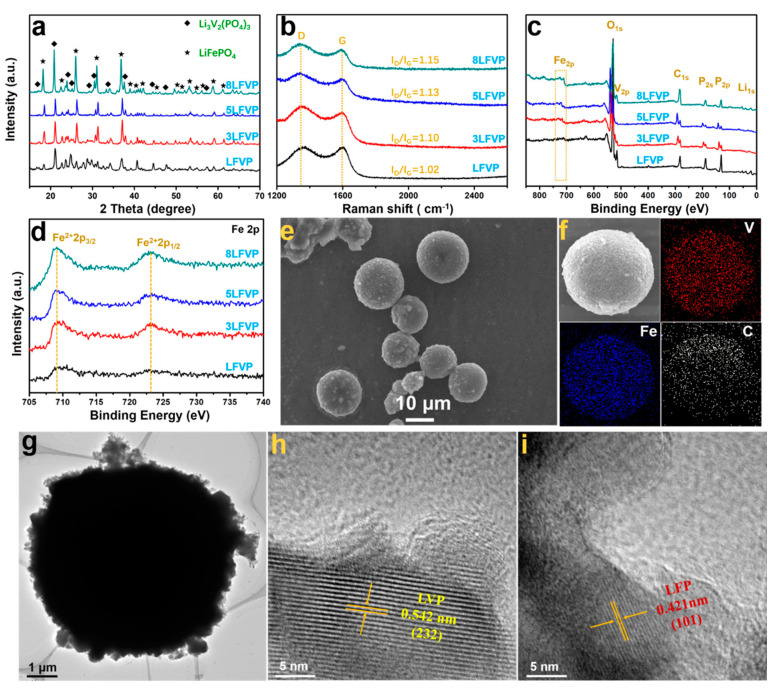
(**a**–**c**) XRD patterns, Raman spectra, and full XPS spectra of the series of xLFP·yLVP composites; (**d**) Fe 2*p* fine spectrum of xLFP·yLVP composites; (**e**,**f**) SEM images of 8LFVP/C with EDS mapping; (**g**) TEM of 8LFVP; (**h**,**i**) HRTEM of 8LFVP with the lattice spacings of LVP and LFP.

Figure 1a shows the XRD patterns of LFVP, 3LFVP, 5LFVP, and 8LFVP. As presented in Figure S1, the diffraction peaks were identified for orthorhombic LiFePO_4_ (JCPDS No. 81-1173) and monoclinic Li_3_V_2_(PO_4_)_3_ (JCPDS No. 01-072-7074) [26,27,28,29]. There were no undesired impurities in this series of composites, indicating the great potential of our adopted material optimization approach in practical application. When comparing all Raman curves of xLFP·yLVP composites in Figure 1b, two apparent peaks near 1350 cm^−1^ and 1590 cm^−1^ could be indexed to D and G bands, respectively, indicating graphite defects and stretching vibration from *sp*^2^ carbon atom bonds [17,30]. However, it should be noted that the graphitization degree of composites showed comparatively subtle differences with increasing LFP content. The I_D_/I_G_ ratio for LFVP was only 1.02, lower than that of 8LFVP (1.15), suggesting that the amorphous carbon embedded on the LVP surface was inclined to the higher electron conductivity, or that vanadium may have had a positive impact on the formation of graphitized carbon. Furthermore, XPS measurement was carried out to obtain the valence state information of key elements in xLFP·yLVP composites. All peaks labeled in Figure 1c represent the index signals for Li 1*s*, P 2*p*, P 2*s*, C 1*s*, V 2*p*, O 1*s*, and Fe 2*p*. Figure 1d presents the XPS curve of Fe 2*p* with two peaks appearing at around 709 eV and 723 eV for Fe 2*p*_3/2_ and Fe 2*p*_1/2_, respectively [31,32]. In particular, the intensity of Fe 2*p* peaks increased gradually with the increase in the raw material proportion of FePO_4_·2H_2_O.

The SEM picture of as-prepared xLFP·yLVP samples in Figure 1e and Figure S2 shows a uniform microsphere structure constructed by our spray-drying technique. The EDS analysis was carried out on the representative selection of 8LFVP, indicating a homogeneous distribution of V, Fe, and C elements in the composites. The TEM and HRTEM measurements provide more detailed information on the structure of 8LFVP, revealing a carbon coating layer outside particles (Figure 1g). Carbon coating has been well documented as an effective modification method for electrode materials with low intrinsic electronic conductivity, allowing for reduced grain growth, enhanced electronic transmission rate, reduced side reaction, and retained structure integrality [14,33,34,35]. Additionally, lattice spacings of 0.542 nm and 0.421 nm were measured, as marked in Figure 1h,i, assigned to the (111) plane of LVP and (101) plane of LFP, respectively.

**Figure 2:**
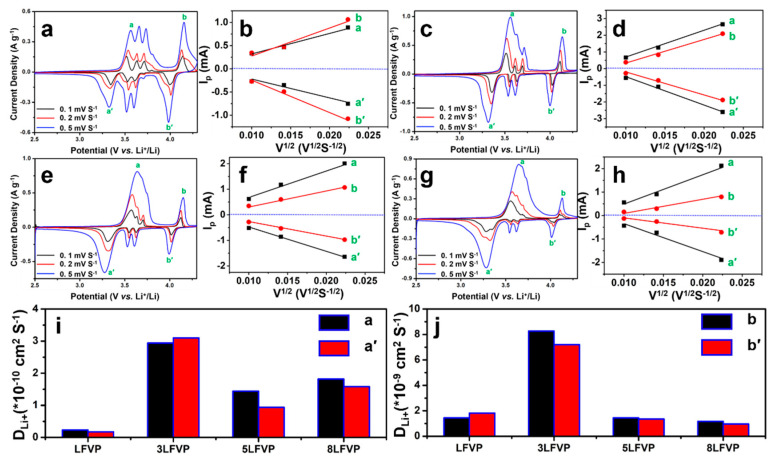
(**a**,**c**,**e**,**g**) CV curves of LFVP, 3LFVP, 5LFVP, and 8LFVP, respectively; (**b**,**d**,**f**,**h**) relationship between I_p_ and V^1/2^ in LFVP, 3LFVP, 5LFVP, and 8LFVP electrodes, respectively; (**i**,**j**) Li-ion diffusion coefficients of LFP and LVP (labeled as a/a′ and b/b′) in series of xLFP·yLVP electrodes.

To determine the differences in intrinsic kinetics, different scan rates of CV measurements were carried out on the series of xLFP·yLVP composites, and the curves are shown in Figure 2a,c,e,g with the potential range of 2.5–4.3 V, corresponding to LFVP, 3LFVP, 5LFVP, and 8LFVP samples, respectively. The redox peak labeled as a/a′ was ascribed to Li-ion reversible extraction/insertion from/into the LFP crystal structure [36,37]. According to previous reports [38,39], the redox reactions for V^3+^/V^4+^ from the LVP electrode can induce three pairs of peak signals, which are shown in the figures except for a/a′. The positions of redox peaks are shifted gradually, associated with enlarged electrochemical polarization, when the scan rates are increased. Furthermore, it is well documented that the electrochemical kinetics is determined by the Li-ion diffusion coefficients, which can be estimated from CV results. Here, the a/a′ and b/b′ redox peaks were chosen to calculate the Li-ion diffusion coefficients (D_Li^+^_) of LFP and LVP, respectively. Figure 2b,d,f,h present the linear fittings of I_p_ vs. v^1/2^, rooting from the Randles–Sevchik equation [34,40]. By calculating the slope of the fitting line, the D_Li^+^_ values of xLFP·yLVP composites were obtained and plotted as a contrast diagram (Figure 2i,j). The D_Li^+^_ of LFP in 3LFVP was 2.94 × 10^−10^/3.1 × 10^−10^ cm^2^·S^−1^, faster than that of other xLFP·yLVP composites. Meanwhile, the D_Li^+^_ of LVP presented similar trend, with 3LFVP having the highest D_Li^+^_ value of 8.27 × 10^−9^/7.2 × 10^−9^ cm^2^·S^−1^. Therefore, it is supposed that 3LFVP could provide better reversibility and the fastest Li-ion migration. However, we should note that the Li-ion diffusion rate in LVP exceeded that in LFP by an order of magnitude, but LFP would deliver higher intrinsic specific capacity. The optimal xLFP·yLVP composition would depend on the specific application requirements.

**Figure 3:**
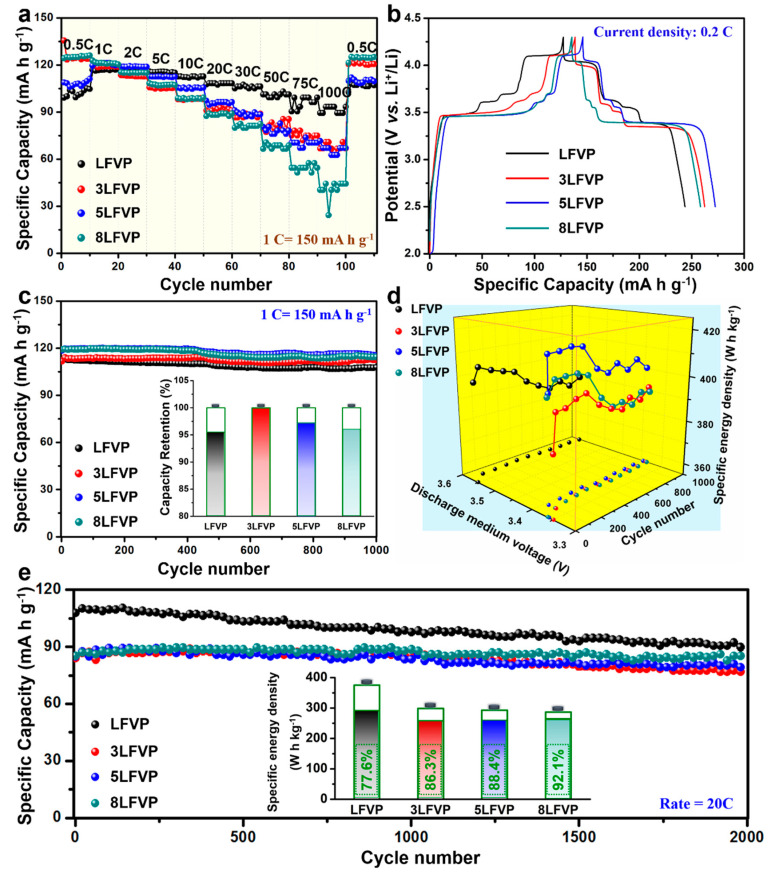
The comparison of xLFP·yLVP electrodes in terms of Li-ion storage capability estimated in the range 2.5–4.3 V; (**a**) rate performance; (**b**) the galvanostatic charge–discharge curves; (**c**) cycling performance at 1 C; (**d**) comparison of the material energy density; (**e**) cycling performance at the high rate of 20 C.

Figure 3a shows the power capabilities of the series of xLFP·yLVP composites, which were evaluated at the step current rate from 0.5 C to 100 C. We can find that, even at the ultrahigh current rate of 100 C, the electrodes could deliver the capacities of 93.3, 70.8, 67, and 44.3 mA·h·g^−1^ for LFVP, 3LFVP, 5LFVP, and 8LFVP electrodes, respectively. Higher specific capacity could be achieved for the 8LFVP electrode at currents less than 2 C. However, when increasing the current rate to 5 C or more, LFVP electrode took the lead due to its high proportion of LVP with a higher ion diffusion coefficient. Figure 3b shows the galvanostatic charge–discharge curves of series of xLFP·yLVP at 0.2 C. It is worth noting that four couples of charge and discharge platforms could be observed for each xLFP·yLVP, agreeing with the results of CV analysis. Moreover, as shown in Figure 3c, when a constant current of 1 C was imposed on each xLFP·yLVP, the initial capacities of 112.7, 111.7, 118.6, and 119.2 mA·h·g^−1^ could be obtained for LFVP, 3LFVP, 5LFVP, and 8LFVP electrodes, respectively. Furthermore, 95.7%, 100%, 97.2%, and 96% capacity retention can be maintained after 1000 cycles, demonstrating excellent structural stability. Although the capacity difference between xLFP·yLVP composites was very small at 1 C, the material energy density varied significantly, which was closely related to discharge medium voltage. As shown in Figure 3d, 5LFVP stood out from the other three electrodes, achieving the highest material energy density of 413.6 W·h·kg^−1^. To acquire more information on the electrochemical capacity of as-prepared xLFP·yLVP electrodes, the ultrahigh rate of 20 C was performed to make a critical assessment of the cyclability (Figure 3e). As expected, the LFVP electrode could deliver a higher capacity of 87.3 mA·h·g^−1^ and an energy density of 291.3 W·h·kg^−1^ after 2000 cycles. Significantly, series of xLFP·yLVP electrodes obtained good energy retentions of 77.6%, 86.3%, 88.4%, and 92.1%. Because of the high power capability of xLFP·yLVP electrodes, our effective material design strategy in this work provides an opportunity for improving the rate performance of LFP in application-oriented Li-ion batteries.

**Figure 4:**
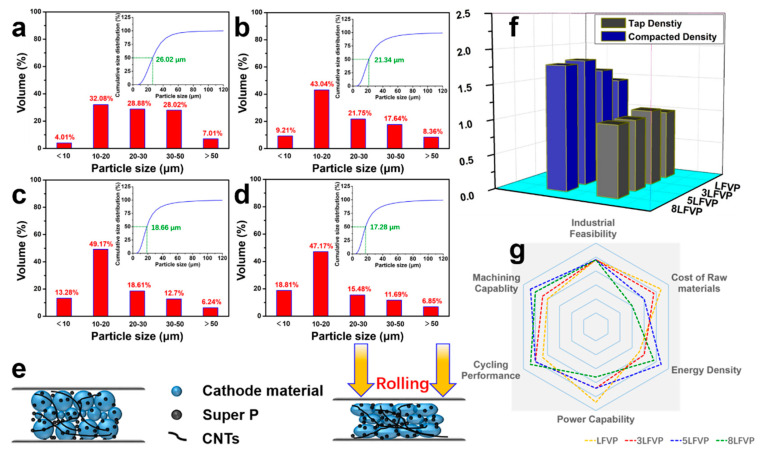
(**a**–**d**) Size distribution of xLFP·yLVP electrode materials, corresponding to LFVP, 3LFVP, 5LFVP, and 8LFVP, respectively; (**e**) illustration of the compaction process of electrode pieces; (**f**) comparison of xLFP·yLVP in terms of tap density for materials and compacted density for electrode pieces; (**g**) compressive evaluation of the series of xLFP·yLVP electrode materials.

Compacted density is a critical parameter to evaluate whether an electrode material can be used in application-oriented Li-ion batteries. Previous reports claimed that high tap density could lead to higher gravimetric and volumetric energy densities. In other words, electrode materials with high tap density would possess high compacted density, which is not entirely precise. In fact, the compacted density can be determined by a variety of factors, involving the components of electrode pieces, the particle size distribution, and the intrinsic true density of electrode material. Hence, here, we applied a laser particle size analyzer to evaluate the size distribution of xLFP·yLVP. As shown in Figure 4a–d, the size range of 10–20 µm occupied a large proportion of the total volume for LFVP, 3LFVP, 5LFVP, and 8LFVP cathode materials, reaching up to 32.08%, 43.04%, 49.17%, and 47.17% of the total, respectively. It is worth noting that 5LFVP with the D_50_ diameter of 18.66 µm demonstrated an optimal size distribution, which may remedy the interstitial space between particles and achieve an enhanced supporting effect [41]. As shown in Figure 4e, 15 tons of pressure imposed on the electrode pieces could compress the invalid space to make close contacts between particles. For the same mass loading, the higher compacted density indicated a thinner electrode and shortened electron pathway. Moreover, Figure 4f attempts to reveal the relation between the compacted and tap density of xLFP·yLVP electrode pieces. 3LFVP provided the highest tap density of 1.08 g·cm^−3^, but a compacted density of only 1.64 g·cm^−3^. The equivalent two values of 5LFVP were 1.03 and 1.82 g·cm^−3^, respectively. According to these results, the tap density of an electrode material does not have a distinct impact on the compacted density of electrode pieces, which can be deeply influenced by size distribution.

A concise evaluation of four types of xLFP·yLVP cathode materials was performed on the basis of six aspects: industrial feasibility, cost of raw material, energy density, power density, cycling performance, and machining capability. As shown in Figure 4g, the increased proportion of LVP in xLFP·yLVP would increase the raw cost, impeding future application in practical Li-ion batteries. Therefore, considering the manufacturing route, electrochemical performance, machining capability, and cost of series of xLFP·yLVP composites, 5LFVP is suggested to be an optimal choice for Li-ion batteries.

Commercialized Li_4_Ti_5_O_12_ with a Spinel structure exhibits superior Li-ion storage capability with long cycling stability at a high rate [42,43], ascribed to the negligible volume change during the Li-ion extraction/insertion process. Therefore, LTO was selected as an anode material in our expected prototype of Li-ion full cell. As shown in Figure S3, commercial LTO with a pure phase exhibited the morphology of block-shaped particles with an average diameter of 1 µm. For achieving a balance between cathode and anode capacity, an LTO||Li half-cell was assembled to estimate the specific capacity and cyclability of LTO electrodes in Figure S4. 

**Figure 5:**
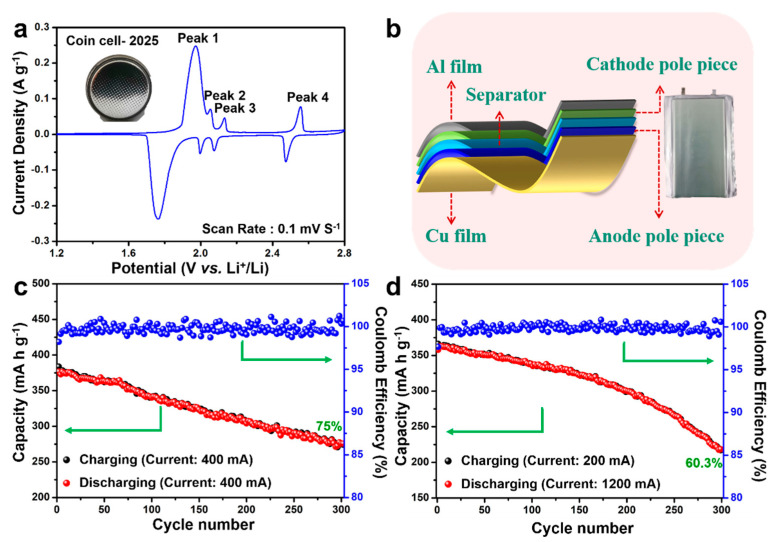
(**a**) CV curves of the 5LFVP||LTO Li-ion full cell measured in the potential range of 1.2–2.8 V; (**b**) schematic of 5LFVP||LTO Li-ion pouch cells; (**c**) cycling performance of 5LFVP||LTO Li-ion pouch cells at the constant current of 400 mA with the capacity retention (**d**) cycling performance of 5LFVP||LTO Li-ion pouch cells under the test conditions of charging at 200 mA and discharging at 1200 mA with the capacity retention.

Here, the Li-ion full battery was assembled with 5LFVP as the cathode and LTO as the anode. The electrochemical behavior of 5LFVP||LTO was analyzed by the CV test, in which the scan rate was set at 0.1 mV·s^−1^. Four couples of redox peaks can be clearly observed from the CV curve in the potential range of 1.2–2.8 V (Figure 5a), owing to the reversible intercalation/deintercalation behavior of Li^+^ ions between the 5LFVP cathode and LTO anode electrodes. It is well documented that assembling Li-ion pouch cells is a more rational way to demonstrate the potential of electrode materials in practical LIBs. As shown in Figure 5b, our 5LFVP||LTO Li-ion pouch cell presented an excellent energy storage capability. At the constant current charge/discharge test of 400 mA (Figure 5c), 75% of the initial capacity (379.1 mA·h) was still achieved after 300 cycles. Significantly, the Coulombic efficiency remained steady at a level of around 100%, indicating great electrochemical reversibility. Moreover, under the test conditions of charging at 200 mA and discharging at 1200 mA (Figure 5d), the initial capacity could reach up to 360.3 mA h. When the cycle number increased to 300 cycles, a capacity retention of 60.3% was recorded for the pouch cell with the capacity fading by 0.15% per cycle. Although the electrochemical performance of 5LFVP||LTO Li-ion pouch cells does not yet meet the requirements for commercialization, this prototype provides a reasonable and scientific attempt for promoting the development of xLFP·yLVP composites in application-oriented Li-ion batteries. 

## 6. Conclusions

In this paper, material engineering optimization was performed to prepare a series of xLFP·yLVP composites using an uncomplicated spray-drying approach, including LFVP, 3LFVP, 5LFVP, and 8LFVP. Taking all the scientific and practical factors into account, 5LFP·LVP stands out as a strong candidate cathode material in Li-ion batteries, attributed to its excellent power capability with a material energy density of 413.6 W·h·kg^−1^, great machining capability with a compacted density of 1.82 g·cm^−3^, and lower raw material cost consumption. Furthermore, the application-oriented energy storage device of the 5LFVP||LTO pouch cell was exploited to demonstrate the Li-ion storage capability. At both low discharge currents of 400 mA and higher currents of 1200 mA, it exhibited superior electrochemical properties. Significantly, the Coulombic efficiency could be maintained around 100% throughout the charge/discharge progress, implying a great electrochemical reversibility. This prototype provides a reasonable and scientific attempt to promote the development of xLFP·yLVP composites in application-oriented Li-ion batteries. 

## Data Availability

Not applicable.

## Supplementary Materials

The following supporting information can be downloaded at https://www.mdpi.com/article/10.3390/ma15103668/s1: Figure S1: The XRD patterns of LFP and LVP, Figure S2. (a–c) SEM images of LFVP/C, 3LFVP/C and 5LFVP/C, respectively; Figure S3. (a) XRD pattern of LTO; (b) SEM image of LTO; (c,d) TEM and HRTEM images of LTO; Figure S4. Li-ion storage capability of LTO estimated in the 0.8–2.2 V; (a) CV curve; (b) The galvanostatic charge–discharge curves at 0.2 C; (c) Rate performance; (d,e) Cycling performance at the rate of 1 and 5 C, respectively; Table S1. The theoretical content of LFP and LVP in xLFP·yLVP composites.
