# Supplementary material for: Material Optimization Engineering toward xLiFePO4·yLi3V2(PO4)3 Composites in Application-Oriented Li-Ion Batteries
Source: Materials (Basel). 2022 May 20;15(10):3668. doi: 10.3390/ma15103668 (PMC9145807; doi:10.3390/ma15103668)
Supplement: Supplementary file 1 [file materials-15-03668-s001.zip › materials-1716263-supplementary.pdf]

Supplementary Materials

Table S1. The theoretical content of LFP and LVP in xLFP·yLVP composites.

	LFVP	3LFVP	5LFVP	8LFVP
**n(LFP):n(LVP)**	**1:1**	**3:1**	**5:1**	**8:1**
**LFP wt%**	**27.90%**	**53.73%**	**65.93%**	**75.59%**
**LVP wt%**	**73.10%**	**46.27%**	**34.07%**	**24.41%**

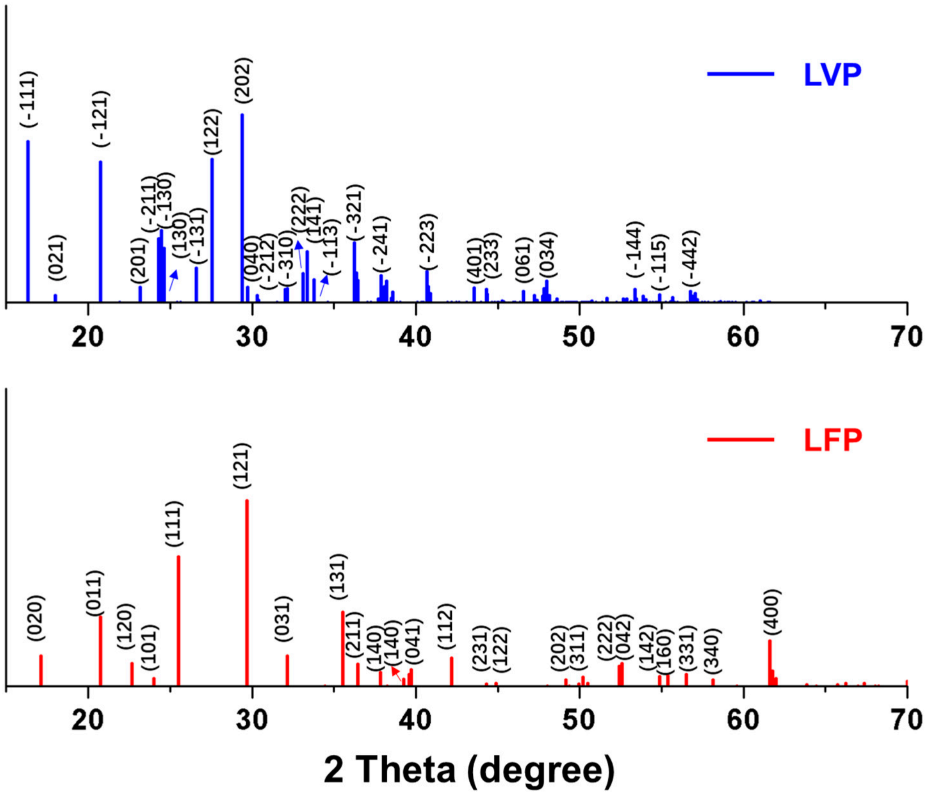

Figure S1. The XRD patterns of LFP and LVP

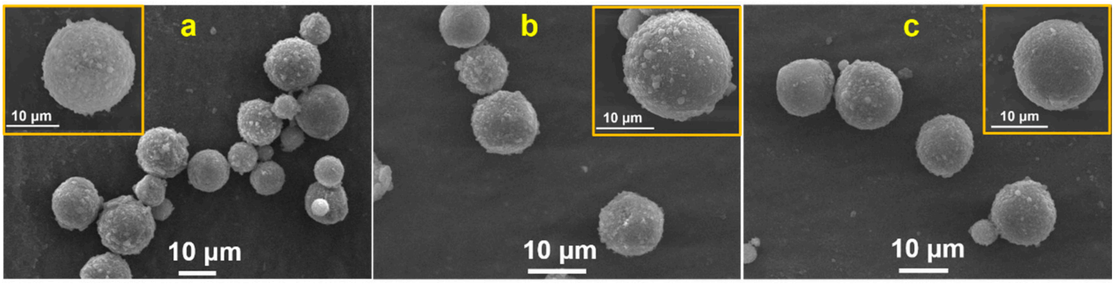

Figure S2. a, b and c) SEM images of LFVP/C, 3LFVP/C and 5LFVP/C, respectively.

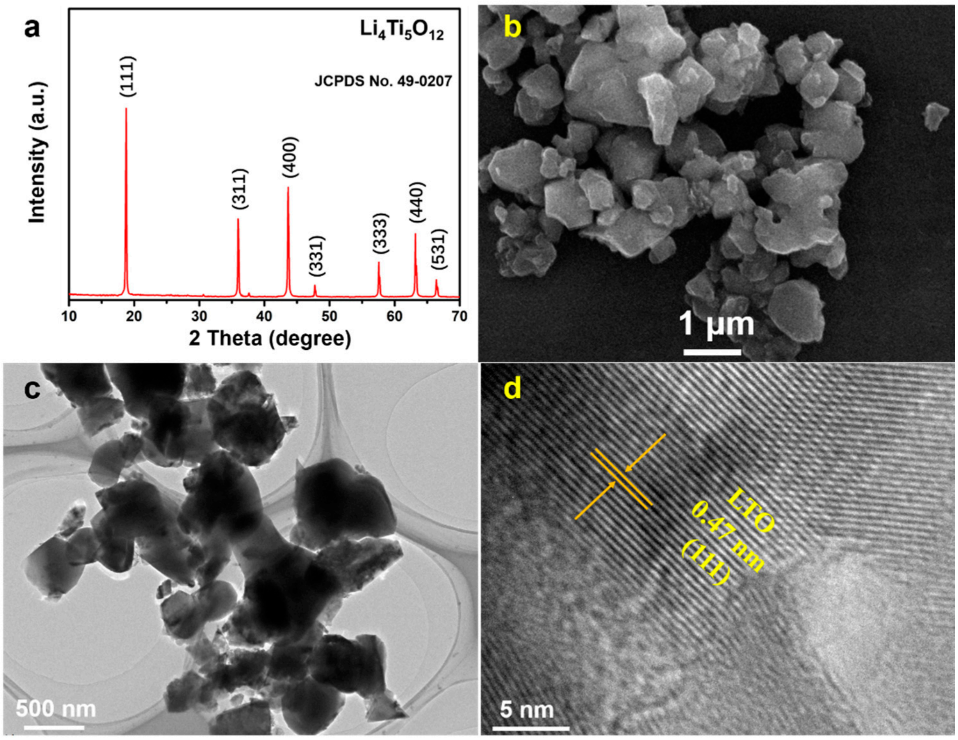

Figure S3. a) XRD pattern of LTO; b) SEM image of LTO; c and d) TEM and HRTEM images of LTO.

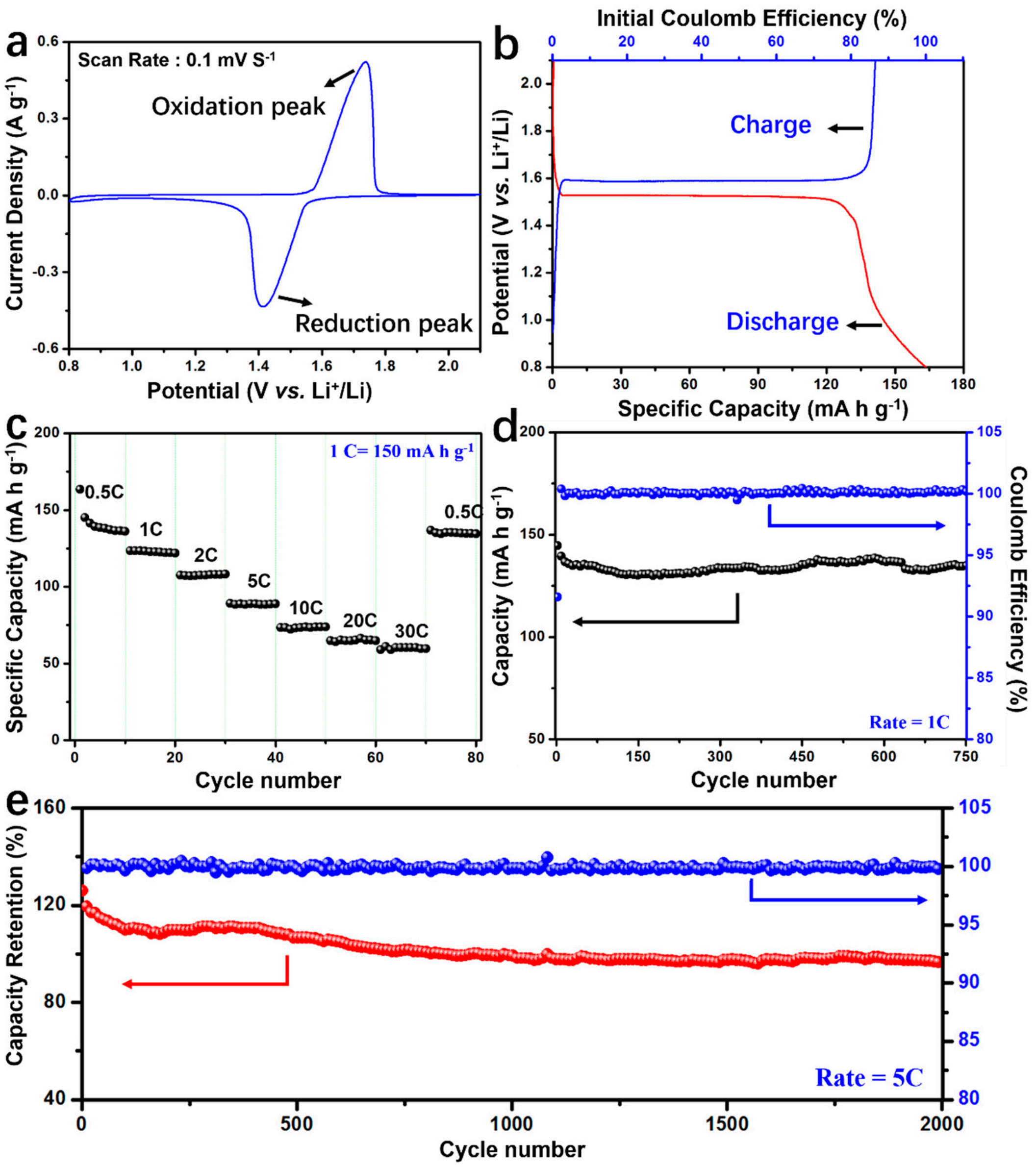

Figure S4. Li-ion storage capability of LTO estimated in the 0.8-2.2 V; a) CV curve; b) The galvanostatic charge–discharge curves at 0.2 C; c) Rate performance; d and e) Cycling performance at the rate of 1 and 5 C, respectively.
